# Association between major depressive episode and risk of type 2 diabetes: A large prospective cohort study in Chinese adults

**DOI:** 10.1016/j.jad.2018.02.052

**Published:** 2018-07

**Authors:** Ruiwei Meng, Na Liu, Canqing Yu, Xiongfei Pan, Jun Lv, Yu Guo, Zheng Bian, Ling Yang, Yiping Chen, Zhengming Chen, An Pan, Liming Li, on behalf of the China Kadoorie Biobank collaborative group

**Affiliations:** a Department of Epidemiology and Biostatistics, Ministry of Education Key Laboratory of Environment and Health, and State Key Laboratory of Environmental Health (Incubating), School of Public Health, Tongji Medical College, Huazhong University of Science and Technology, Wuhan, China; b Department of Nutrition and Food Hygiene, School of Public Health, Tongji Medical College, Huazhong University of Science and Technology, Wuhan, China; c Department of Epidemiology and Biostatistics, School of Public Health, Peking University Health Science Center, Beijing, China; d Chinese Academy of Medical Sciences, Beijing, China; e Clinical Trial Service Unit & Epidemiological Studies Unit (CTSU), Nuffield Department of Population Health, University of Oxford, United Kingdom

**Keywords:** Major depressive episode, Type 2 diabetes, Prospective cohort study, Chinese

## Abstract

**Objective:**

We aimed to prospectively evaluate the association between major depressive episode (MDE) and risk of type 2 diabetes in a Chinese population.

**Methods:**

We used data from the China Kadoorie Biobank study, in which 461,213 participants free of diabetes, coronary heart disease, stroke, and cancer were followed from baseline (2004–2008) to December 31, 2013. A modified Chinese version of Composite International Diagnostic Interview Short-Form (CIDI-SF) was used to assess past year MDE. Participants who responded positive to depression screening questions but did not meet the diagnosis criteria were considered as having depressive symptoms only. Incident diabetes cases were identified through linkage with established regional disease registries and national health insurance databases. Cox proportional hazards regression model was used to calculate hazard ratio (HR) and 95% confidence interval (CI) for the association after adjusting for diabetes risk factors.

**Results:**

We documented 8784 incident type 2 diabetes cases during a follow-up of 3291,908 person-years. We observed a higher incidence rate of type 2 diabetes in participants with MDE than those without, and the multivariable-adjusted HR was 1.31 (95% CI 1.04–1.66). Compared with participants without depressive symptoms, the HR (95% CI) was 1.19 (1.05–1.35) for participants with depressive symptoms only and 1.32 (1.05–1.68) for those with MDE.

**Limitations:**

The prevelance of past year MDE based on CIDI-SF was low, which might result in a selection bias.

**Conclusions:**

In our large prospective cohort study of Chinese adults, MDE was significantly and independently associated with an increased risk of type 2 diabetes.

## Highlights

• We examined the depression-diabetes relationship in a large cohort of Chinese adults of almost half million participants.
• Major depressive episode was associated with about 30% higher risk of type 2 diabetes.
• The depression-diabetes relationship might be dose-response in Chinese adults.

## 1 Introduction

Diabetes and depression are two common and major non-communicable diseases with significant disease burdens worldwide. According to the Global Burden of Disease Study 2016, diabetes and depression caused more than 57 and 34 million, respectively, all-age disability-adjusted life-years (DALYs) in 2016 (GBD 2016 DALYs and HALE Collaborators, 2017). The International Diabetes Federation estimated that 1 in 11 adults (415 million) had diabetes in 2015 and the number was projected to be 642 million by 2040 if the rising trend continues (International Diabetes Federation, 2016). Meanwhile, the global point prevalence and the annual incidence rate of major depressive disorder was estimated to be 4.7% and 3.0%, respectively (Ferrari et al., 2013, Kessler et al., 2015). In China, diabetes and depression are also major public health challenges. A recent national survey reported that the estimated prevalence of diabetes in adults was 10.9% in 2013 (Wang et al., 2017), which translated to 118 million people with diabetes. In addition, 35.7% (388 million) of adults had prediabetes (Wang et al., 2017), highlighting the importance and urgency of diabetes prevention in China. Meanwhile, a recent systemic analysis reported that 2.2% of men and 3.3% of women in China suffered from major depressive disorder (Baxter et al., 2016). Depression caused more than 10 million DALYs in 2013 in China, and this number would increase by 11% in men and 8% in women by 2025 (Charlson et al., 2016).

Growing evidence suggests that depression is associated with an increased risk of incident diabetes, as summarized in several meta-analyses (Knol et al., 2006, Rotella and Mannucci, 2013; M. Yu et al., 2015). For example, a recent meta-analysis of 23 cohort studies reported a pooled relative risk of 1.38 (95% confidence interval [CI] 1.23–1.55) (Rotella and Mannucci, 2013). However, most of the studies were performed in Western populations, and few studies have explored this association in Chinese or even Asian populations. We only found one prospective study on this topic in 2380 male employees of an electrical company in Japan, which reported a positive association between self-reported depressive symptoms and risk of incident diabetes (Kawakami et al., 1999). However, the sample size of this study was small with only 41 incident diabetes cases occurred in 8 years of follow-up. There are a few cross-sectional studies in Chinese population. One cross-sectional study based on the Taiwan Health Insurance database in 2005 reported that individuals with clinically-diagnosed major depressive disorder had a significant higher prevalence of diabetes than the general population (Chien et al., 2012). We have previously shown in the baseline data of the China Kadoorie Biobank (CKB) study that self-reported major depression was significantly higher among participants with clinically-identified type 2 diabetes but not screen-detected type 2 diabetes compared to those without type 2 diabetes (Mezuk et al., 2013). However, the temporal relation may not be easily illustrated in cross-sectional studies, and the comorbidity could be because depression increases risk of diabetes or vice versa. Therefore, we aimed to examine the prospective association between major depression and risk of type 2 diabetes in the large population-based cohort of Chinese adults.

## 2 Methods

### 2.1 Study population

Our study was based on data collected from the CKB study. Detailed information on the design, survey methods, population, and follow-up of the CKB study has been described elsewhere (Chen et al., 2011). A total of 512,891 individuals (59% women) aged 30–79 years were recruited during 2004–2008 from 10 diverse areas across China (five urban areas and five rural areas) and were followed up to December 31, 2013 in our study. These areas were selected based on socioeconomic status, diversity of exposures and disease patterns, stability of the population and local infrastructures, and long-term local commitment. The estimated population response rate was about 30% (26–38% in the five rural areas and 16–50% in the five urban areas) (Chen et al., 2011). Before the study started, a detailed data collection protocol was developed in Chinese by experts from the Oxford University, local, regional and national Centers for Disease Control and Prevention of China, as part of a robust training program for the field workers and interviewers. The manual thoroughly explained the interpretation and wording of each question. The interviewers were required to explain each question and answer option using standard text from the manual. Furthermore, to safeguard the quality of data collection, we routinely took audio recordings of the interviews, in order to identify mistakes at an early stage, and to provide additional training or replace underperformed staff.

At baseline, trained investigators interviewed each participant using a standardized laptop-based questionnaire to collect socio-demographic characteristics, lifestyle factors, personal and family medical history, and mental health status. The laptop-based questionnaire had built-in logic and error checks to ensure valid and reliable input of answers. The CKB study was approved by research ethics committees at the Oxford University, national and local Centers for Disease Control and Prevention (CDC) in China, and written informed consent was obtained from each participant.

In this study, we excluded participants with major chronic diseases at baseline to reduce the potential reverse causation. Individuals who reported a history of diabetes (self-reported or fasting glucose ≥ 7.0 mmol/L or random glucose ≥ 11.1 mmol/L, n= 30,300), cancer (n = 2577), coronary heart diseases (CHD, n = 15,472), stroke or transient ischemic attack (n = 8884), and who had no information of BMI (n = 2) at baseline were excluded. A total of 461,213 participants (59% women) were included for the current analysis. The study flow was shown as Supplemental Fig. 1. All individuals provided information on the past year depression status.

### 2.2 Major depressive episode assessment

Past year major depressive episode was evaluated at baseline using the modified Chinese version of Composite International Diagnostic Interview Short-Form (CIDI-SF) (Kessler et al., 1998). The sensitivity and specificity of CIDI in Chinese population was reported to be 69.6% and 96.7%, respectively, in a validation study (Lu et al., 2015). The CIDI-SF takes a shorter time to complete but achieves relatively high overall classification accuracy of major depressive episode compared to the full CIDI (Kessler et al., 1998), which is a structured diagnostic instrument designed from the Diagnostic and Statistical Manual of Mental Disorders IV (Kessler and Ustun, 2004).

First, participants were screened for the question whether they had the following symptoms for more than two consecutive weeks in the past 12 months: 1) more sad or more depressed than usual; 2) loss of interest in most things that usually give pleasure such as hobbies or activities; 3) feeling hopeless and having no appetite for favorite food; and 4) feeling worthless and useless, blaming for anything that went wrong, or feeling that life was difficult and there was no way out. If they answered “yes” to any of the four questions, they were then further interviewed by trained health workers using the CIDI-SF. Past year major depressive episode was diagnosed if they responded positively to the screening question, and had 3 or more out of 7 diagnostic symptoms, which including loss of interest and pleasure, fatigue or loss of energy, weight change, sleep problems, concentration problems, feelings of worthlessness, and thoughts of suicide. We also conducted an ad hoc analysis by further classifying participants as having depressive symptoms only for those who answered “yes” to any of the screening questions but did not fulfill the diagnostic criteria, and categorizing individuals who responded “no” to the screening question as having no depressive symptoms.

### 2.3 Follow-up and type 2 diabetes ascertainment

All study participants were followed for mortality and morbidity by the CKB Regional Coordinating Centre Office. Vital status of study participants was periodically renewed based on the China's Disease Surveillance Points, checked annually against local residential records and health insurance records. Information on incident diseases (mainly including cancer, ischemic heart disease, stroke, and diabetes) or hospital admission was collected through linkage with regional disease registries and health insurance systems using the national citizen identification number. Incident diseases were coded according to the International Statistical Classification of Diseases and Related Health Problems 10th Revision (ICD-10) by trained staff who were blinded to the baseline information (Chen et al., 2011).

The primary endpoint in our analysis was incident type 2 diabetes (ICD-10 code E11 and E14). According to findings from the outcome adjudication study for diabetes during 2012 and 2013, which rechecked original medical records and laboratory results of 831 randomly selected diabetes cases, 98.6% of them were reconfirmed (Lv et al., 2017).

### 2.4 Assessment of covariates

The covariates were obtained from interviewer-administered questionnaires at baseline, including demographic or socioeconomic characteristics (age, sex, geographic location, education, household income, and marital status), lifestyle factors (alcohol use, smoking status, physical activity, and intakes of red meat, fresh fruits and vegetables), previous psychiatric comorbidities (psychiatric disorders and neurasthenia disorders) and hypertension, and family history of diabetes. Metabolic equivalent of task per day (METs-h/day) spent on both work and leisure activities was used to quantify the physical activity level (Du et al., 2013). Physical measurements, including height, weight, and blood pressure, were conducted by trained health workers using standard instruments and protocols. BMI was defined as the weight in kilograms divided by the square of the height in meters. Prevalent hypertension was diagnosed as systolic blood pressure ≥ 140 mm Hg, or diastolic blood pressure ≥ 90 mm Hg, or self-reported diagnosis of hypertension or taking antihypertensive drugs at baseline.

### 2.5 Statistical analyses

Baseline characteristics are presented according to depression status as means ( ± SD) for continuous variables, or percentages (%) for categorical variables and they were compared using Student *t*-test or χ^2^ test, respectively. Entry time was defined as the date of baseline interview and censor time was defined as the date of type 2 diabetes diagnosis, death, loss to follow-up, or December 31, 2013, whichever came first. Cox proportional hazards regression models were used to calculate hazard ratios (HRs) and 95% CIs for the associations, with adjustment for baseline covariates and with stratification by baseline age (in 5-year intervals), sex and survey region. The proportional hazards assumption for the Cox model was checked by adding an interaction term of follow-up duration and the exposure in the model and no violation was found. Stepwise multivariable models were used to adjust for potential confounders, which were selected on previous studies of the association between depression and type 2 diabetes risk and *a priori* knowledge of underlying biological mechanisms (Hu et al., 2001, Lv et al., 2017). We also conducted stratified analyses by sex, age at baseline (≥ 60 or < 60 years old), BMI (≥ 24 or < 24 kg/m^2^) to investigate whether the pre-specified factors modified the association. Statistical tests for interactions were conducted by adding a two-way interaction term between major depressive episode and the stratified factor (both as binary variables) in the final model. In order to investigate the possibility of dose-response relationship, we conducted the ad hoc analysis of classifying the participants into three categories: no depressive symptoms (reference group), depressive symptoms only, and major depressive episode. In order to minimize the possibility of reverse causality, i.e., undiagnosed or subclinical type 2 diabetes leading to depressive episode, we conducted a sensitivity analysis by excluding participants who developed type 2 diabetes during the first two years of follow-up (n = 1383). To avoid the influence of other psychiatric comorbidities on the association between past year major depressive episode with risk of type 2 diabetes, sensitivity analyses were conducted by excluding participants with diagnosed psychiatric disorders (n = 1641) and/or neurasthenia disorders (n = 4714) at baseline. We also included participants with baseline cancer, coronary heart disease, and stroke in a sensitivity analysis to evaluate the robustness of the results, and those baseline comorbidities were further adjusted in the final model. All analyses were performed using SAS version 9.3 (SAS Institute, Cary, NC, USA) and statistical significance was based on 2-side test at the 0.05 significance level.

## 3 Results

The overall 12-month prevalence of major depressive episode was 0.61% (n = 2801) in 461,213 Chinese adults. Baseline characteristics of the individuals were shown in Table 1 according to depression status. Compared to participants without major depressive episode, those with major depressive episode were younger, and more likely to be women and solitary (widowed, separated/divorced, or never married), and to have lower levels of education, total household income, BMI, red meat and fresh fruits consumptions, but were less likely to be physically active, to smoke cigarettes or drink alcohol, or have hypertension. The prevalence of psychiatric disorders and neurasthenia was higher in participants with compared to those without major depressive episode. No significant difference was found for fresh vegetable intake and family history of diabetes between the two groups.

**Table 1:** Baseline characteristics of participants according to major depressive episode status.

Characteristics	Total (n = 461,213)	Major depressive episode status		*P* value*
		Yes (n = 2801)	No (n = 458,412)	
Age (years)	50.7 ± 10.5	50.2 ± 9.9	50.7 ± 10.5	< 0.001
BMI (kg/m^2^)	23.5 ± 3.3	23.0 ± 3.3	23.5 ± 3.3	< 0.001
Total physical activity (MET-hours/day^a^, %)	21.9 ± 13.9	21.0 ± 14.3	21.9 ± 13.9	< 0.001
Sex (%)				< 0.001
Men	189,154 (41.0)	798 (28.5)	188,356 (41.1)	
Women	272,059 (59.0)	2003 (71.5)	270,056 (58.9)	
Education level, n (%)				< 0.001
No formal school	85,240 (18.5)	602 (21.5)	84,638 (18.5)	
Primary	148,253 (32.1)	999 (35.7)	147,254 (32.1)	
Middle school	131,881 (28.6)	756 (27.0)	131,125 (28.6)	
High school	69,802 (15.1)	333 (11.9)	69,469 (15.1)	
College/university or more	26,037 (5.7)	111 (3.9)	25,926 (5.7)	
Household income (RMB/year, %)				< 0.001
< 5000	45,150 (9.8)	525 (18.7)	44,625 (9.7)	
5000–9999	86,585 (18.8)	597 (21.3)	85,988 (18.8)	
10,000–19,999	132,888 (28.8)	808 (28.9)	132,080 (28.8)	
≥ 20,000	196,590 (42.6)	871(31.1)	195,719 (42.7)	
Marital status, n (%)				< 0.001
Married	420,124 (91.1)	2092 (74.7)	418,032 (91.2)	
Widowed	30,341 (6.6)	538 (19.2)	29,803 (6.5)	
Separated/divorced	7217 (1.5)	129 (4.6)	7088 (1.5)	
Never married	3531 (0.8)	42 (1.5)	3489 (0.8)	
Survey region, n (%)				< 0.001
Qingdao	30,516 (6.6)	54 (1.9)	30,462 (6.6)	
Harbin	45,154 (9.8)	338 (12.1)	44,816 (9.8)	
Haikou	27,017 (5.8)	38 (1.4)	26,979 (5.9)	
Suzhou	49,266 (10.7)	368 (13.1)	48,898 (10.7)	
Liuzhou	42,907 (9.3)	133 (4.8)	42,774 (9.3)	
Sichuan	52,865 (11.5)	345 (12.3)	52,520 (11.5)	
Gansu	47,068 (10.2)	231 (8.2)	46,837 (10.2)	
Henan	57,281 (12.4)	428 (15.3)	56,853 (12.4)	
Zhejiang	53,916 (11.7)	325 (11.6)	53,591 (11.7)	
Hunan	55,223 (12.0)	541 (19.3)	54,682 (11.9)	
Smoking status, n (%)				< 0.001
Never	28,5497 (61.9)	1928 (68.8)	283,569 (61.9)	
Occasional	26,526 (5.8)	167 (6.0)	26,359 (5.7)	
Former	24,671 (5.3)	97 (3.5)	24,574 (5.4)	
Current	124,519 (27.0)	609 (21.7)	123,910 (27.0)	
Drinking status, n (%)				< 0.001
Never regular	209,002 (45.3)	1458 (52.0)	207,544 (45.3)	
Ex-regular	7066 (1.5)	62 (2.2)	7004 (1.5)	
Occasional or monthly or reduced intake	174,943 (37.9)	994 (35.5)	173,949 (38.0)	
Weekly or more	70,202 (15.2)	287 (10.3)	69,915 (15.2)	
Red meat, n (%)				< 0.001
Daily	132,324 (28.7)	536 (19.1)	131,788 (28.7)	
4–6 days per week	84,252 (18.2)	405 (14.5)	83,847 (18.3)	
1–3 days per week	164,956 (35.8)	1142 (40.8)	163,814 (35.7)	
Monthly	58,090 (12.6)	497 (17.7)	57,593 (12.6)	
Never/rarely	21,591 (4.7)	221 (7.9)	21,370 (4.7)	
Fresh vegetables, n (%)				0.052
Daily	436,453 (94.6)	2643 (94.3)	433,810 (94.6)	
4–6 days per week	16,697 (3.6)	100 (3.6)	16,597 (3.6)	
1–3 days per week	6626 (1.4)	47 (1.7)	6579 (1.4)	
Monthly	1327 (0.3)	8 (0.3)	1319 (0.3)	
Never/rarely	110 (0.1)	3 (0.1)	107 (0.1)	
Fresh fruits, n (%)				< 0.001
Daily	83,767 (18.2)	351 (12.5)	83,416 (18.2)	
4–6 days per week	43,844 (9.5)	195 (7.0)	43,649 (9.5)	
1–3 days per week	147,012 (31.9)	825 (29.5)	146,187 (31.9)	
Monthly	159,149 (34.5)	1116 (39.8)	158,033 (34.5)	
Never/rarely	27,441 (5.9)	314 (11.2)	27,127 (5.9)	
Family history of diabetes, n (%)				0.41
Yes	21,228 (4.6)	138 (4.9)	21,090 (4.6)	
No	439,985 (95.4)	2663 (95.1)	437,322 (95.4)	
Baseline history of hypertension, n (%)				< 0.001
Yes	144,103 (31.2)	780 (27.9)	143,323 (31.3)	
No	317,110 (68.8)	2021 (72.1)	315,089 (68.7)	
Baseline history of psychiatric disorder, n (%)				< 0.001
Yes	1641 (0.4)	133 (8.1)	2668 (0.6)	
No	459,572 (99.6)	1508 (91.9)	456,904 (99.4)	
Baseline history of neurasthenia, n (%)				< 0.001
Yes	4714 (1.0)	175 (6.2)	4539 (1.0)	
No	456,499 (99.0)	2626 (93.8)	453,873 (99.0)	

Data are shown as mean ± standard deviation for continuous variables, or n (%) for categorical variables.

* *P* values were calculated by Student *t*-test for continuous variables or χ^2^ test for categorical variables.

a MET-hours/day: metabolic equivalent of task value for a day's work and leisure activities for both farmers and non-farmer.

A total of 8784 incident type 2 diabetes cases were identified during a median follow-up of 7.2 years (Table 2). The incidence density rate of type 2 diabetes was 3.36 per 1000 person-years in individuals with major depressive episode versus 2.66 per 1000 person-years in those without major depressive episode. Past year major depressive episode was associated with a 24% increase in the hazard of developing type 2 diabetes with adjustment for age, sex, geographic location, marital status, education level, total household income, and lifestyle factors, but this was not statistically significant (HR 1.24, 95% CI 0.98–1.57). However, after further adjustment for BMI, hypertension status, and family history of diabetes, the HR increased to 1.31 and was statistically significant (95% CI 1.04–1.66). No significant interactions were found with age, sex, or BMI categories (Table 2).

**Table 2:** Major depressive episode status and risk of incident type 2 diabetes.

MDE status	Cases/person-years	Incidence rate (1000 person-years)	Model 1	Model 2	Model 3	*P* values for interaction
			HR (95% CI)	HR (95% CI)	HR (95% CI)	
Total						
No MDE	8714/3271,064	2.66	1	1	1	
MDE	70/20,844	3.36	1.24 (0.98–1.57)	1.24 (0.98–1.57)	1.31 (1.04–1.66)	
Sex						0.39
Men						
No MDE	3245/1330,966	2.44	1	1	1	
MDE	14/5839	2.40	1.09 (0.65–1.85)	1.06 (0.63–1.80)	1.09 (0.64–1.85)	
Women						
No MDE	5469/1940,097	2.82	1	1	1	
MDE	56/15,005	3.73	1.28 (0.99–1.67)	1.28 (0.99–1.67)	1.38 (1.06–1.79)	
Age						0.62
<60 years old						
No MDE	5923/2597,289	2.28	1	1	1	
MDE	51/17,254	2.96	1.29 (0.97–1.69)	1.28 (0.97–1.69)	1.38 (1.04–1.82)	
≥ 60 years old						
No MDE	2791/673,776	4.14	1	1	1	
MDE	19/3590	5.29	1.15(0.73–1.80)	1.15 (0.73–1.81)	1.19 (0.76–1.87)	
BMI						0.91
<24 kg/m^2^						
No MDE	3072/1894,523	1.62	1	1	1	
MDE	30/13,323	2.25	1.36 (0.94–1.94)	1.35 (0.94–1.94)	1.39 (0.97–1.99)	
≥24 kg/m^2^						
No MDE	5642/1376,542	4.1	1	1	1	
MDE	40/7521	5.32	1.28 (0.94–1.75)	1.27 (0.93–1.74)	1.27 (0.93–1.73)	

Note: MDE, major depressive episode; HR, hazard ratio; CI, confidence interval.

Model 1: adjusted for age, sex, geographic location, marital status, education, and household income.

Model 2: model 1 plus smoking status, drinking status, physical activity, red meat consumption frequency, vegetable consumption frequency, and fruit consumption frequency.

Model 3: model 2 plus body mass index, history of hypertension, and family history of diabetes.

In addition, compared with those with no depressive symptoms, participants with depressive symptoms only had a 19% (HR 1.19, 95% CI 1.05–1.35) increased risk of type 2 diabetes, and those with major depressive episode had a 32% (HR 1.32, 95% CI 1.05–1.68) increased risk of type 2 diabetes (*P* for trend < 0.001; Table 3).

**Table 3:** Association between depressive symptoms and risk of incident type 2 diabetes.

	No depressive symptoms	Depressive symptoms only	Major depressive episode	*P* for trend
Cases/person-years	8466/3195,788	248/75,276	70/20,844	_
Incidence rate (1000 person-years)	2.65	3.29	3.36	_
Model 1	1	1.11 (0.98–1.26)	1.25 (0.99–1.58)	0.02
Model 2	1	1.13 (0.99–1.28)	1.25 (0.98–1.58)	0.01
Model 3	1	1.19 (1.05–1.35)	1.32 (1.05–1.68)	<0.001

Model 1: adjusted for age, sex, geographic location, marital status, education, and household income.

Model 2: model 1 plus smoking status, drinking status, physical activity, red meat consumption frequency, vegetable consumption frequency, and fruit consumption frequency.

Model 3: model 2 plus body mass index, history of hypertension, and family history of diabetes.

In the sensitivity analyses, the association between major depressive episode and risk of type 2 diabetes was not materially changed after excluding incident diabetes cases diagnosed within the first two years of follow-up (HR 1.31, 95% CI 1.02–1.70), or excluding participants with baseline psychiatric disorders (HR 1.32, 95% CI 1.04–1.68), or additional exclusion of those with neurasthenia disorders (HR 1.27, 95% CI 0.99–1.64; Supplementary Table 1). In addition, significant association was also found when including those with baseline history of cancer, coronary heart disease and stroke in the analysis (HR 1.34, 95% CI 1.07–1.67; Supplementary Table 2).

## 4 Discussion

In this large cohort study of Chinese adults, we observed that individuals with major depressive episode had a 31% increased risk of type 2 diabetes compared to those without major depressive episode. The association was independent of other baseline covariates which were established or potential risk factors of diabetes, including sociodemographic and lifestyle factors, family history and baseline comorbidities. In addition, compared to those without depressive symptoms, participants with depressive symptoms also had a significantly increased risk of type 2 diabetes, although the effect estimate was much lower than those with major depressive episode. Our findings add support to the current evidence that depression is a risk factor for type 2 diabetes.

In the present study, we found a statistically significant, though modest, elevation in risk of type 2 diabetes with a multivariable-adjusted HR of 1.31, which is in accordance with the results of a previous meta-analysis that reported a pooled relative risk of 1.38 (95% CI 1.23–1.55) (Rotella and Mannucci, 2013). Although many studies have reported the association between depression and incident diabetes, most of them were conducted in US (n = 11) or Europe (n = 11), and only one study in Asians (Japanese) in this meta-analysis. In this Japanese study (Kawakami et al., 1999), depression was evaluated by the Japan version of Zung Self-Rating Depression (SDS). A total of 41 incident diabetes cases were identified from 2380 male workers during the 8 years’ follow-up. A positive association was reported between moderate or severe levels of depressive symptoms and onset of type 2 diabetes (HR 2.31, 95% CI 1.03–5.20) after controlling for the potential diabetes risk factors. Therefore, to our best knowledge, our study is the first to assess the relationship between depression and incident diabetes in Chinese population. We also found a stronger relationship in women than men (HR, 1.38 versus 1.09), although without significant effect modification (*P* for interaction = 0.39). Further studies are needed to explore whether there are any sex differences in the depression-diabetes association.

So far, our study was the largest one in this field with approximately 0.5 million participants. In the present study, we included 461,213 participants without type 2 diabetes at baseline and documented 8784 incident type 2 diabetes cases during a median of 7.2 years’ follow-up. The latest meta-analysis on this topic included 33 studies (24 cohorts, 2 nested case-control studies, 7 cross sectional studies) with 2411,641 individuals (M. Yu et al., 2015). Our results are consistent with several previous large cohort studies in Caucasians (Golden et al., 2004), although some studies did not found a significant association (Knol et al., 2007). For example, the US Atherosclerosis Risk in Communities Study included 11,615 individuals aged 45–64 years, in which the results showed that elevated depressive symptoms was related to a 31% increased risk of type 2 diabetes (multivariate-adjusted HR 1.31, 95% CI 1.01–1.64) (Golden et al., 2004). However, another large cohort in 42,426 adults from the Netherlands did not find a significant association between depression and diabetes (HR 1.06, 95% CI 0.89–1.26) (Knol et al., 2007). While another study in the Dutch population found a significant association between current depressive disorders defined by CIDI and 2-year risk of type 2 diabetes, although the sample size was small with 2460 participants and 31 incident diabetes cases (Renn et al., 2011).

In many previous studies, depression was assessed by questionnaires of self-reported symptoms only and few studies used clinical diagnostic schedule. In our study, after a few screening questions, depressive symptoms were assessed by modified Chinese version of CIDI-SF. Although cannot be considered equivalent to the gold standard, the CIDI-SF has been shown to produce an acceptable sensitivity (89.6%) and specificity (93.9%) compared to the full-version CIDI (Kessler et al., 1998). A number of studies have reported a dose-response relation between depression severity and diabetes risk (Golden et al., 2004, Kawakami et al., 1999). We have also shown in our analysis that participants with major depressive episode had a much higher risk of incident diabetes, while those with depressive symptoms only were at a relatively lower increased risk compared to those without depressive symptoms. However the classification of “depressive symptoms only” group was arbitrary and needed to be validated in future studies. A few studies have suggested that the relation between depression and diabetes could be bidirectional (Atlantis et al., 2012), and diabetes was also related to an increased risk of depression. Therefore, to reduce the possibility of reverse causation (individuals with subclinical or asymptomatic diabetes developed depression), we excluded type 2 diabetes cases diagnosed within the first 2 years after recruitment, and the results persisted in this sensitivity analysis.

The association between depression and increased risk of type 2 diabetes was complicated and there were several potential mechanisms underlying this relationship, of which pathophysiological changes and unhealthy lifestyle habits were the major concerns. On the one hand, depression was associated with hyperactivity of hypothalamic-pituitary-adrenal (HPA) axis and sympathetic nervous system, resulting in increased release of counterregulatory hormones which contributed to abdominal adiposity and insulin resistance (Knol et al., 2006; M. Yu et al., 2015). Inflammatory markers were associated with both depression (Raison et al., 2006) and development of diabetes (Pradhan et al., 2001), thus increased inflammation might be a biological pathway between depression and onset of diabetes (Knol et al., 2006). On the other hand, depressed patients often had unhealthy habits, including smoking, physical inactivity, excess calorie intake, substance or alcohol abuse, and nonadherence with medications (Knol et al., 2006, Rotella and Mannucci, 2013; M. Yu et al., 2015). These unhealthy habits were often risk factors of diabetes (Hu et al., 2001, Lv et al., 2017). However, adjustment for these risk factors in the present study did not attenuate the association. In our study, the association was strengthened in the final model after further adjustment for baseline BMI, indicating that BMI might be a major reverse confounder. In our study, the BMI levels were lower in people with depression compared to those without depression. In some previous cross-sectional studies of Chinese and Korean populations, depression was inversely associated with obesity and the “jolly fat” hypothesis has been proposed (Kim et al., 2010, Knol et al., 2007). Additionally, certain anti-depressant drugs have been reported to increase risk of type 2 diabetes (Pan et al., 2012, Rubin et al., 2008), and could also be a part of the biological mechanisms. However, we did not collect the information on anti-depressant and could not test this hypothesis in our study. But only 15% of the depressed individuals in CKB sought helps from doctors (C. Yu et al., 2015), and thus the influence from anti-depressant drugs could be small.

Our study has several strengths, including the large-scale population-based sample, a relatively long term and high follow-up rate, the validated and structured diagnostic instrument for depression measurements, and detailed information on diabetes risk factors and potential confounders. However, several limitations should also be acknowledged. First, a selection bias may be present. The prevelance of past year major depressive episode (0.61%) based on CIDI-SF was much lower than several studies reported previously in Chinese and Western populations. For example, a cross-sectional survey in a sample of 63,004 Chinese adults reported that the adjusted 1-month prevalence of major depressive disorder was 2.1% (Phillips et al., 2009); another earlier study in Beijing and Shanghai reported that the estimated 1-year prevalence of major depressive episode using CIDI was 1.8% in the 5201 adults (Lee et al., 2009); and according to the results of the US National Comorbidity Survey Replication, the 12-month prevalence of major depressive disorder was 6.6% in adults aged ≥ 18 years (Kessler et al., 2003). The variation in the depression prevalence across studies might result from different instruments used to assess depression, different sampling framework and culture backgrounds. In our study, depression was assessed by general health workers while the previous study in 63,004 Chinese adults measured depression through a semi-structured interview performed by professional psychiatric nurses (Phillips et al., 2009). In addition, stigma of depression and mental health is common in Chinese traditional culture and individuals might deny or minimize their experience of depression. Furthermore, healthy volunteers were more likely to accept invitation to participate in a cohort study like CKB, while people with severe depression might not be willing to take part in a cohort study because they were not interested in most things. All of these factors could lead to a lower detection rate and relatively lower depression prevalence. However, our study was not designed to provide a representative estimate of depression prevalence in the general population, but to evaluate the temporal relation between depression and incident diabetes. If individuals with more severe depression were less likely to participate in the study, the included depression cases would comprise more people with moderate symptoms and the incidence rate of diabetes in this group would be lower than expected, and the true depression-diabetes association would be underestimated. Furthermore, we only assessed the past year MDE and did not know the life-time depression status. Thus, the observed association could be underestimated because some participants without past year MDE may have depression in the past and were also at higher risk of diabetes (Bruce et al., 2016).

Second, in our study, we only measured the past year depression status once at baseline and did not know the dynamic changes during the follow-up or the duration of depression over time. In addition, the exact information of inflammatory markers and anti-depressant medication use was not available in our study, and it needs further investigation whether inflammatory makers or anti-depressant use could explain the observed association. Third, screening bias cannot be excluded as a possible explanation of the association. Participants with depression may frequently visit doctors and have blood glucose measured. However, only about 15% of the depressed individuals sought help from doctors in the CKB study (C. Yu et al., 2015); therefore, we deemed that the influence of screening bias should be small. Fourth, the baseline diabetes was defined by self-reported diabetes diagnosis or medication use, fasting glucose or random glucose in the CKB study, and the incident diabetes was identified through linkage with regional disease registries and health insurance systems. Therefore, the baseline diabetes prevalence was lower than the national survey result which additionally included 2-h glucose tolerance test and hemoglobin A1c measures (Wang et al., 2017), and the incidence rate was also low given that many asymptomatic diabetes in China were undiagnosed if no blood test was given (Wang et al., 2017). Such non-differential misclassification might lead to attenuation of effect estimates. Finally, although we have controlled for various established diabetes risk factors in our study, residual confounding is still possible.

## 5 Conclusions

In conclusion, we found a positive association between past year major depressive episode and risk of type 2 diabetes in this well-established mega-cohort study of Chinese adults. The association was independent of other major diabetes risk factors. Given the rapidly increased disease burden of depression and diabetes in China, our study has a profound importance for both clinical practice and public health policies. In China, many people with depression do not seek help from doctors and do not get recommended treatment (C. Yu et al., 2015). Those people have a higher risk of diabetes and other chronic diseases such as ischemic heart disease and stroke as demonstrated in previous publications from CKB (Liu et al., 2016, Sun et al., 2016; C. Yu et al., 2015). Future studies are still needed to validate our findings in Chinese population and explore the potential mechanisms underlying the association. Clinicians who treat patients with depression should be aware of the potential increased risk of diabetes and closely monitor the glucose and metabolic factors in those patients. Finally, more researches are also needed to evaluate whether treatment of major depressive episode (such as behavioral modification and drug treatment) and alleviation of depressive symptoms could ameliorate metabolic abnormality in high risk individuals and eventually reduce the risk of diabetes in Chinese adults.
