# Supplementary material for: Association between major depressive episode and risk of type 2 diabetes: A large prospective cohort study in Chinese adults
Source: J Affect Disord. 2018 Jul;234:59–66. doi: 10.1016/j.jad.2018.02.052 (PMC5909035; doi:10.1016/j.jad.2018.02.052)
Supplement: Supplementary file 1 — Supplementary material. [file mmc1.docx]

**Association between major depressive episode and risk of type 2 diabetes: a large prospective cohort study in Chinese adults**

Meng R, Liu N, Yu C, Pan X, Jun L, Guo Y, Bian Z, Yang L, Chen Y, Wu T, Chen Z, Pan A, Li L, on behalf of the China Kadoorie Biobank collaborative group

| **Supplementary Table 1**  Association between major depressive episode and risk of type 2 diabetes: sensitivity analysis. | | | | | |
| --- | --- | --- | --- | --- | --- |
|  | Cases/person-years | Incidence rate (1000 person-years) | Model 1 | Model 2 | Model 3 |
|  |  |  | HR (95% CI) | HR (95% CI) | HR (95% CI) |
| Sensitivity analysis 1 | |  |  |  |  |
| No MDE | 7,341/3,269,449 | 2.25 | 1 | 1 | 1 |
| MDE | 60/20,832 | 2.88 | 1.24 (0.96-1.61) | 1.24 (0.96-1.60) | 1.31 (1.02-1.70) |
| Sensitivity analysis 2 | |  |  |  |  |
| No MDE | 8,668/3,260,390 | 2.66 | 1 | 1 | 1 |
| MDE | 67/19,861 | 3.37 | 1.24 (0.98-1.58) | 1.31 (1.03-1.66) | 1.32 (1.04-1.68) |
| Sensitivity analysis 3 | |  |  |  |  |
| No MDE | 8,546/3,228,235 | 2.65 | 1 | 1 | 1 |
| MDE | 60/18,672 | 3.21 | 1.20 (0.93-1.55) | 1.26 (0.97-1.62) | 1.27 (0.99-1.64) |

MDE, major depressive episode; HR, hazard ratio; CI, confidence interval.

Sensitivity analysis 1: excluding cases occurring within the first 2 years of follow-up.

Sensitivity analysis 2: excluding those with baseline psychiatric disorder.

Sensitivity analysis 3: excluding those with baseline psychiatric disorders and neurasthenia disorder.

Model 1: adjusted for age, sex, geographic location, marital status, education, and household income.

Model 2: model 1 plus smoking status, drinking status, physical activity, red meat consumption frequency, vegetable consumption frequency, and fruit consumption frequency.

Model 3: model 2 plus body mass index, history of hypertension, and family history of diabetes.

| **Supplementary Table 2**  Association between major depressive episode and risk of type 2 diabetes: including participants with cancer, CHD, or stroke. | | | | | |
| --- | --- | --- | --- | --- | --- |
|  | Cases/person-years | Incidence rate (1000 person-years) | Model 1 | Model 2 | Model 3 |
|  |  |  | HR (95% CI) | HR (95% CI) | HR (95% CI) |
| No MDE | 9,412/3,413,010 | 2.75 | 1 | 1 | 1 |
| MDE | 81/22,492 | 3.60 | 1.29 (1.04-1.61) | 1.28 (1.03-1.62) | 1.34 (1.07-1.67) |

MDE, major depressive episode; CHD, coronary heart diseases; HR, hazard ratio; CI, confidence interval.

Model 1: adjusted for age, sex, geographic location, marital status, education, and household income.

Model 2: model 1 plus smoking status, drinking status, physical activity, red meat consumption frequency, vegetable consumption frequency, and fruit consumption frequency.

Model 3: model 2 plus body mass index, history of hypertension, cancer, CHD, stroke, and family history of diabetes.

**Supplementary Figure 1** Study flow


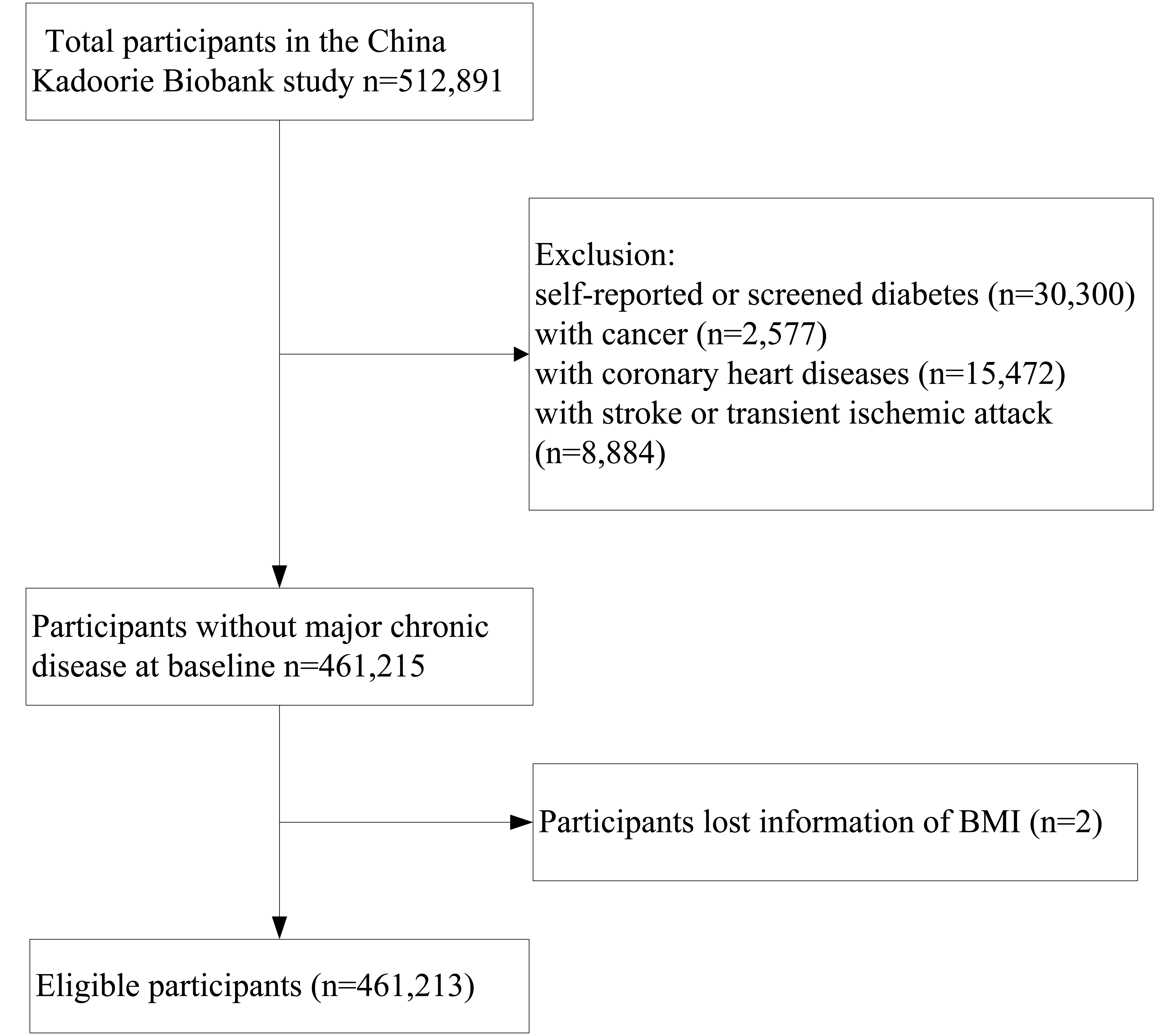


Figure legend

There was a total of 5,557 participants who met more than 1 exclusion criterion.
